# Prediction of open urinary tract in laparoscopic partial nephrectomy by virtual resection plane visualization

**DOI:** 10.1186/1471-2490-14-47

**Published:** 2014-06-13

**Authors:** Daiki Ueno, Kazuhide Makiyama, Hiroyuki Yamanaka, Takashi Ijiri, Hideo Yokota, Yoshinobu Kubota

**Affiliations:** 1 Department of Urology, Yokohama City University School of Medicine, 3-9 Fukuura, Kanagawa-ku, Yokohama, Kanagawa 236-0004, Japan; 2 Image Processing Research Tea, RIKEN, Wako, Japan

**Keywords:** Laparoscopic surgery, Partial nephrectomy, Simulator, Urinary tract opening

## Abstract

**Background:**

The purpose of this study is presenting a method to predict the presence of an open urinary tract and the position of the opening in laparoscopic partial nephrectomy from three dimensional (3D) computed tomography (CT) images by using novel image segmentation and visualization techniques.

**Methods:**

From CT images of patients who underwent laparoscopic partial nephrectomy, 3D regions of the kidney, urinary tract, and tumor were segmented. For each patient, multiple virtual resection planes of the kidney with different surgical margins (1 mm to 5 mm, every 1 mm) were generated and the presence of an open urinary tract and the position of the opening were predicted from the images.

**Results:**

We compared the predictions with actual operations in 5 cases by using recorded video of the operations and operative notes. In terms of the presence of an open urinary tract, agreement of the predictions and the intraoperative results was obtained in all patients. The expected positions of the openings were close to those in the actual operations.

**Conclusions:**

We have developed a method to virtually visualize the resection plane of laparoscopic partial nephrectomy. Image segmentation methods used in this study were precise and effective. The comparison indicated that our method accurately predicted the presence of an open urinary tract and the position of the opening and provided useful preoperative information.

## Background

Laparoscopic surgery has been preferred by many surgeons and patients because of shorter recovery time and less surgical invasion [1]. A partial nephrectomy shows treatment outcomes equal to those of a radical nephrectomy and preserves postoperative renal function [2-4]. Therefore, laparoscopic partial nephrectomy has become an increasingly common practice for small renal tumors.

To achieve a safe procedure, preoperative information is especially important for laparoscopic surgery. Organs and large vessels around the operated site are difficult to recognize only with the limited two dimensional (2D) view of the laparoscope. It is important to preoperatively understand the anatomical features of the patient.

We developed the three dimensional (3D) image processing software VoTracer to preoperatively obtain significant information from computed tomography (CT) or magnetic resonance imaging (MRI) images. VoTracer supports traditional volume rendering; the user can observe 3D images by modifying local transparency and changing the viewpoint [5]. It also supports various image segmentation methods, such as thresholding [6], region growing [7], graph cut [8,9], and contour-based segmentation [10,11]; the user can quickly segment organ and tumor regions from 3D images with simple interaction.

One of the postoperative complications of partial nephrectomy is urine leakage. If the urinary tract is open during tumor resection, suturing the crack is required for the prevention of postoperative urine leakage. To formulate an operation strategy, it is useful to predict the margin of the urinary tract opening during the tumor resection.

In this study, we focused on laparoscopic partial nephrectomy and presented a method to predict the presence of an open urinary tract and the position of the opening using VoTracer. We also evaluated our method by comparing our predictions with actual operation results.

## Methods

### Patients and characteristics

This was a retrospective study including 5 patients who underwent laparoscopic partial nephrectomy at Yokohama City University Hospital by a single surgeon (K.M.) between April 2011 and January 2013. We created virtual resection plane images on another day by accessing medical archives and evaluated it. The study protocol was approved by the Yokohama City University Institutional Review Board. Written informed consent was obtained from all patients. Application of laparoscopic partial nephrectomy was limited to cases where the tumor size was 4 cm or less and the operation could be performed safely. The surgical margin and the angle of cut in were determined by full observation of the tumor by intraoperative ultrasound. Tumor resection was performed by cold cut using scissors with kidney ischemia. Then partial nephrectomy is performed. After the retrograde injection of dilute indigo carmine, the continuous suturing of the opened collecting system and transected major vessel is performed by 2–0 Vicryl on a SH needle with an intracorporeal knot-tying.

### CT image segmentation and virtual-resection-plane

From the delay phase of enhanced CT images of each patient, we segmented three regions: kidney, urinary tract, and tumor. Different segmentation methods were applied for each region. We then generated a virtual resection plane of the kidney by using segmentation. The whole process was performed via VoTracer.

**Figure 1:**
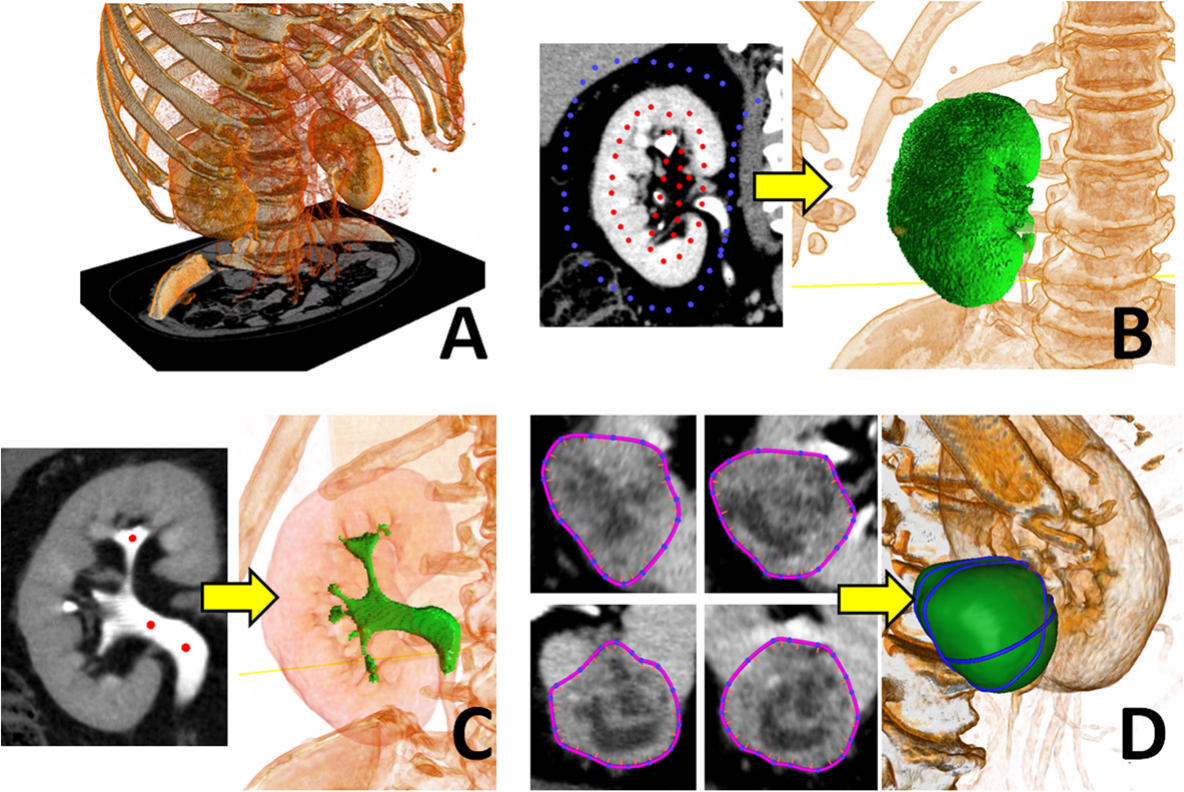
**Segmentation of CT image.** From an input 3D CT image **(A)**, we segmented the three regions by different methods. **(B)** The kidney region was segmented by the graph cut method [8,9]. We specified several foreground (red) and background (blue) points on a cross section and the method computed the kidney region from the points. **(C)** The urinary tract region was segmented by region growing [7]. The method grew the urinary tract region from several seeds (red points). **(D)** The tumor region was segmented by the contour-based method [11]. The method computed the boundary surface from specified multiple contours.

The graph cut image segmentation method [8,9] was applied to segment the kidney region. We roughly specified inside and outside control points on several cross sections of the 3D CT images and the graph cut algorithm segmented the kidney region accurately (Figure 1B). The region growing method [7] was used for the urinary tract. We specified several seeds in the urinary tract region and the method evolves a region from the seeds. We computed the evolution only in voxels of which CT values were larger than *r*, where *r* ∈ [350, 420] was a parameter selected depending on the patient (Figure 1C). The contour-based 3D image segmentation method [11] was employed for segmenting the tumor. We specified several contours on the tumor in the 3D images and the method optimized a segmentation boundary that passed through all contours and had a smooth shape (Figure 1D). This method was useful for regions that did not have obvious high-contrast boundaries.

**Figure 2:**
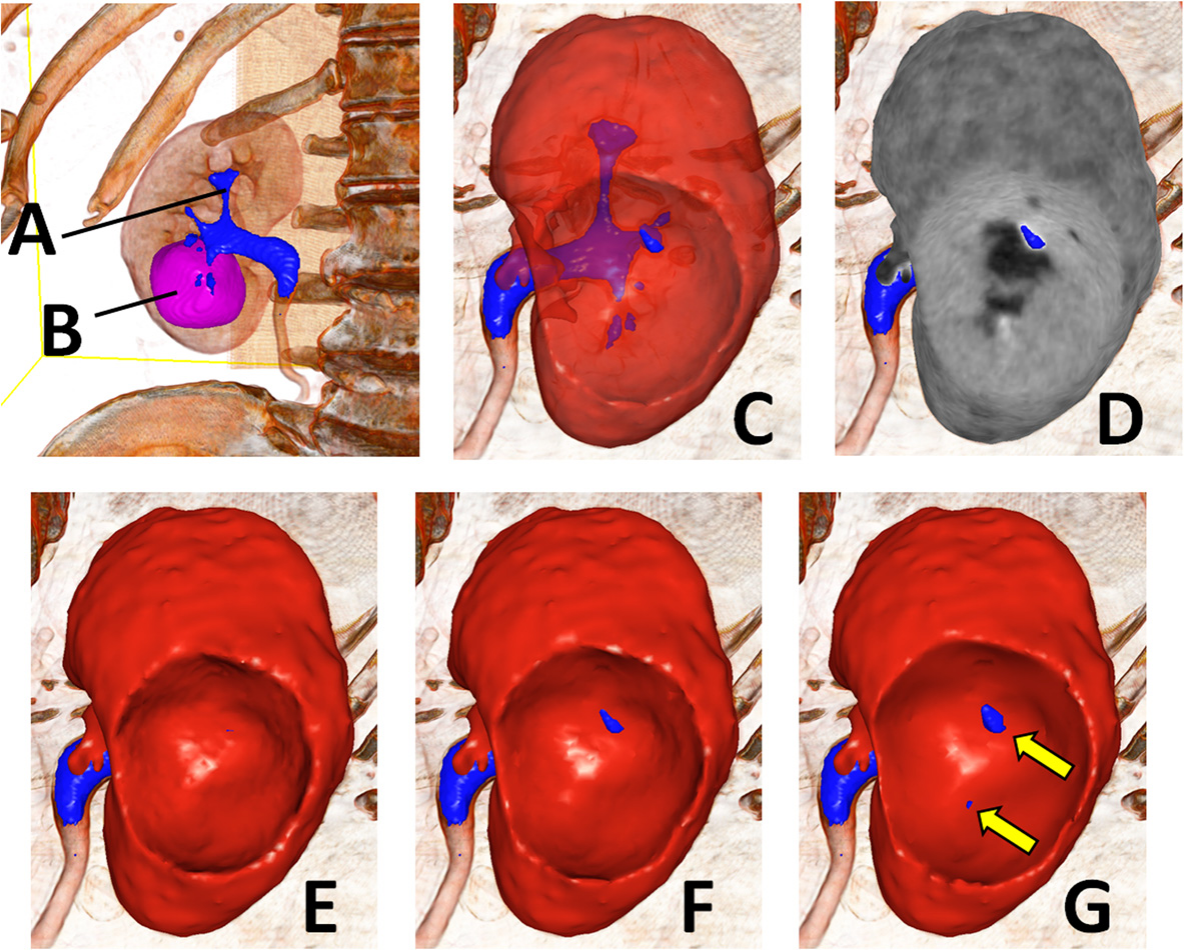
**Virtual resection plane.** We generated surface models of the urinary tract **(A)** and tumor **(B)** regions. We also generated multiple kidney regions that had 1 mm **(E)**, 3 mm **(F)**, and 5 mm **(G)** margins from the tumor. We also generated transparent **(C)** and CT-image renderings **(D)** of the kidney model. The CT-image rendering **(D)** allowed observance of the CT value on the resection plane.

Given the image segmentation, we generated a virtual resection plane. We first generated polygon surface models of the kidney, urinary tract, and tumor regions by Marching cubes algorithm [12] (Figure 2). We then deformed the kidney model so that it had a certain margin from the tumor (Figure 2E-G). We also visualized the CT images on the kidney surface as in Figure 2D. The virtual resection planes visually provided information on the presence of an open urinary tract and the position of the opening in partial nephrectomy.

### Prediction and evaluation

A single evaluator (D.U.), who did not know the details of the operation, performed the segmentation of the 3D CT images of the 5 patients using VoTracer. A software developer (T.I.) supported the segmentation process. For each patient, the evaluator created virtual resection planes with 1 mm to 5 mm margins, every 1 mm margin, and predicted whether and where the urinary tract was open during the actual operation (Figure 2).

We compared the predictions and intraoperative results. The presence of an open urinary tract during the actual operation was verified from recorded video of the operation and operative notes. In cases where there was an open urinary tract, the positions of the openings were compared with those in the virtual resection planes.

## Results

**Figure 3:**
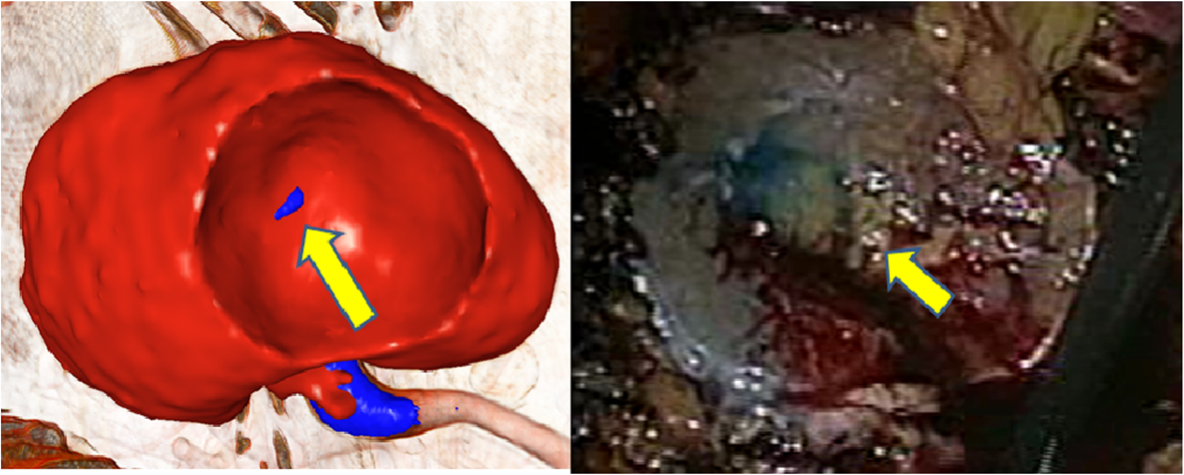
**Side-by-side comparison.** The right panel is a representative scene from a recorded video of an operation and the left panel shows a virtual resection plane similar to the viewpoint of the actual operation.

Table 1 summarizes the backgrounds of the patients, prediction results, and surgical outcomes. Table 1 also shows the minimum margins of the openings the evaluator detected in the virtual resection planes and actual surgical margins measured from the operation samples at the bottom of the tumor.In terms of presence of an open urinary tract (i.e. minimum opening margin), the predictions and intraoperative results approximately coincided in all patients. However, in patient No. 2, the predicted opening margin was 1 mm greater than the actual margin, and this may be explained by the size of the margin, which was likely to be larger at the site of urinary tract opening than at the bottom. The margin is generally the smallest at the bottom, and becomes larger at the periphery. We think this small difference was not critical for predicting the possible presence of an open urinary tract. The positions of the openings were similar to the predicted positions (Figure 3). In the two cases where the urinary tract was open, the patients were properly treated and no patient had postoperative complications. Surgical pathology reported clear cell renal cell carcinoma with negative margins in all cases.

**Table 1:** Patient characteristics and summary of the result

**No**	**Sex**	**Age**	**Tumor size (mm)**	**Tumor location**				**Prediction of urinary tract status**	**Intraoperative urinary tract status**	**Surgical margin (min)**	**Ischemia time (min)**	**Operating time (min)**
				**Right/Left**	**Longitudinal location**	**Arterior/Posterior**	**Lateral/Medial**					
1	M	44	19	R	Upper pole	Anterior	Lateral	Not open with 5 mm margin	Not open	3	19	185
2	F	77	26	L	Upper pole	Anterior	Medial	Open with 2 mm margin	Open	1	20	179
3	M	72	35	R	Inferior pole	Posterior	Lateral	Open with 1 mm margin	Open	1	28	206
4	M	65	19	R	Middle pole	Posterior	Medial	Not open with 5 mm margin	Not open	1	13	156
5	M	75	25	R	Middle pole	Posterior	Medial	Open with 4 mm margin	Not open	1	11	158

In the columns, from left to right, we indicate patient information, tumor size, tumor location, prediction of urinary tract status, intraoperative urinary tract status, intraoperative surgical margin, ischemia time, and operating time.

## Discussion

In this study, we focused on open urinary tracts and developed a way to virtually visualize resection planes from CT images. Preoperatively understanding the 3D relationship of organs is particularly important for laparoscopic partial nephrectomy. In the past, surgeons had to infer the relationship of organs from CT slices; they performed 3D shape reconstructions in their heads. This required extensive experience and it was impossible to share ideas with others. In contrast, our method provides visualization that is close to the actual intraoperative view and allows surgeons to easily understand the 3D relationship of important organs. We believe that the preoperative information provided by our visualization method improves the safety of operations.

The major postoperative complications of laparoscopic partial nephrectomy are urine leakage, parenchymal bleeding and acute renal failure. Breda et al. reported that urine leakage of laparoscopic partial nephrectomy occurred in 1.4-2.0% of patients [13]. Thompson et al. reported that ischemia time is the most important factor in predicting renal function preservation [14]. To prevent complications, cracks should be securely sutured and ischemia time should be decreased, which can be accomplished by appropriate preoperative planning. For instance, the approach (trans-abdominal or trans-retroperitoneum), port position, and resection line should be selected depending on the tumor position and surrounding organs. Gill et al. reported on the novel technique of zero ischemia partial nephrectomy with preoperative planning using a visualized 3D view of the renal artery [15]. Our method virtually visualizes the intraoperative view from a different viewpoint. It may allow surgeons to visualize operation procedures in a different preoperative setting and enable them to select a better setting. Nephrometry scoring systems have been developed in an effort to standardize tumor assessment. R.E.N.A.L. Nephrometry Score (NS) has been proposed by Uzzo et al. [16]. Ficarra et al. introduced Preoperative Aspects and Dimensions Used for an Anatomical (PADUA) system. Our visualization technique contributes to more precise scoring of those nephrometry scores [17]. Our study focused on visualization of open urinary tracts, the first such study to have been reported.

Training for laparoscopic surgery is much more difficult than that for open surgery. Since laparoscopic surgery has a steep learning curve, it is important to provide sufficient training. Various laparoscopic surgery training systems have been developed. Some recent systems reproduces the entire procedure of laparoscopic radical nephrectomy [18,19]. However, to the best of our knowledge, no other system provides an actual operative view of the individual patient. In contrast, our method provides visualization similar to the actual laparoscopic operative view using individual patient data.

The image segmentation software VoTracer, which we developed for this study, is useful in creating simulation models. Our research group has published studies on a mission rehearsal type surgical simulator that uses patient-specific models [20-22]. In these studies, we reported that the patient-specific surgical simulator is useful for less-experienced doctors in practicing standard operations and for experts in developing new surgical procedures. However, one of the biggest obstacles of such a simulator is the process of creating simulation models from 3D images of patients. The image segmentation method presented in this study allowed us to segment each region (kidney, urinary tract, and tumor) in a few minutes. This drastically accelerates the model creation process. Combining the surgical simulator and the image segmentation method is an ongoing project that we hope to realize in the future.The major limitation of our current method is that the segmentation process requires user operation. The user has to set region growing parameters to correctly segment the urinary tract region. The user also has to specify contours to segment tumor regions based on his or her subjective knowledge (Figure 1D). Thus, segmentation results may vary depending on the user. Notice that this is retrospective study and the number of cases is also restricted. Larger population study is required in order to raise the accuracy of prediction. In the future, we would like to conduct a large-scale study and develop a segmentation method that minimizes user operation as much as possible. In this study, we used the delay phase of contrast enhanced CT in order to precisely segment the urinary tract. Due to CT imaging timing issues, we had several cases in which it was difficult to precisely segment the urinary tract. It is necessary to determine the best timing of CT imaging in order to stably segment urinary tract regions.

## Conclusions

In this study, we developed a method to predict the presence of an open urinary tract and the position of the opening in laparoscopic partial nephrectomy from 3D CT images. The results revealed that we could make accurate predictions in 5 cases. This was a retrospective study and the number of cases was small. A larger scale study is required in order to confirm the accuracy of the predictions.

## Abbreviations

3D: Three dimensional; 2D: Two dimensional; CT: Computed tomography; MRI: Magnetic resonance imaging; NS: Nephrometry score.

## Competing interests

The authors declare that they have no competing interests.

## Authors’ contributions

DU made conception and desingn and drafted the manuscript. KM participated in the design of the study, and revised the manuscript. HY collected and assemble the data. TI and HY developed the software used in this study. YK conceived of the study, and helped to draft the manuscript. All authors read and approved the final manuscript.

## Pre-publication history

The pre-publication history for this paper can be accessed here:

http://www.biomedcentral.com/1471-2490/14/47/prepub
